# Exosomal hsa_circ_0008925 from Urine Is Related to Chronic Renal Fibrosis

**DOI:** 10.1155/2022/1899282

**Published:** 2022-02-18

**Authors:** Yuhan Cao, Yuanhui Shi, Yuwei Wang, Yanlang Yang, Wenjun Guo, Cuifeng Zhang, Wenjun Pei, Cong Fu

**Affiliations:** ^1^Department of Nephrology, Yijishan Hospital Affiliated to Wannan Medical College, Wuhu, China; ^2^Anesthesia Laboratory and Training Center of Wannan Medical College, Wuhu, China; ^3^Key Laboratory of Non-coding RNA Transformation Research of Anhui Higher Education Institution (Wannan Medical College), Wuhu, China; ^4^Department of Anesthesiology, Yijishan Hospital Affiliated to Wannan Medical College, Wuhu, China; ^5^Anhui Province Key Laboratory of Biological Macromolecules Research (Wan Nan Medical College), Wuhu, China; ^6^Department of Cardiology, Yijishan Hospital Affiliated to Wannan Medical College, Wuhu, China

## Abstract

At present, there is no noninvasive biomarker of renal fibrosis. The potential diagnostic value of urinary exosome-derived circRNAs from glomerular disease patients for renal fibrosis is still uncertain. Here, we first detected the expression of hsa_circ_0008925 in TGF-*β*1-cultured HK-2 cell-derived exosomes. Secondly, we collected urine samples from 95 biopsy-proven glomerular disease patients and 34 healthy controls. The expression of hsa_circ_0008925 was analyzed, and the correlation with renal function and pathological changes was calculated. The receiver operating characteristic (ROC) curve for the diagnosis of renal fibrosis was performed. The results showed that in exosomes derived from TGF-*β*1-cultured HK-2 cells, the expression of hsa_circ_0008925 was increased compared with normal cultured. Further, the expression level of hsa_circ_0008925 was increased in urinary exosomes from renal fibrosis patients and correlated with serum creatinine, blood urea nitrogen (BUN), estimated glomerular filtration rate, and cystatin C. The level of hsa_circ_0008925 was furthermore correlated with the score of tubulointerstitial fibrosis (TIF) and the score of glomerular sclerosis. The ROC curve showed that hsa_circ_0008925 can diagnose renal fibrosis at a cut-off value of 0.093 with a sensitivity of 52.2% and specificity of 96.4%. In summary, we indicated that urinary exosomal hsa_circ_0008925 could be acted as a noninvasive biomarker for renal fibrosis in glomerular diseases patients.

## 1. Introduction

Chronic kidney disease (CKD) is a major medical problem in China and around the world. A cross-sectional survey revealed that the morbidity of CKD in China is 10.8% [1]. Glomerular disease is a common type of CKD. Glomerular disease includes many pathological types. Renal fibrosis (RF) which is defined as glomerulosclerosis and tubulointerstitial fibrosis (TIF) is a common outcome of all pathological types [2]. RF is a typical manifestation of end-stage renal disease. Renal biopsy is the golden standard for the diagnosis of renal fibrosis. Renal biopsy is a risky procedure that cannot be used as a routine examination in glomerular disease patients. Previous studies have found that urine-containing molecules have the potential to serve as noninvasive biomarkers for RF [3, 4]. So far, there is still no method for the noninvasive and dynamic detection of RF that can be applied in clinical practice.

The biological information contained in urine as well as urinary exosomes has great potential to become biomarkers for glomerular disease and renal fibrosis. Real-time quantitative polymerase chain reaction- (qPCR-) based urinary RNA detection has been found as a novel method for detection of biomarkers of kidney disease [5, 6]. Recently, noncoding RNA such as circRNAs showed great aptitude as biomarkers of kidney disease progression [7–9]. Urine also contained exosomes [10]. And exosomes contained proteins and RNA, from the cytoplasm of the cells [11, 12]. Urinary exosome secretion and their content, especially mRNAs, miRNAs, and circRNAs, were considered the potential candidates for novel biomarkers of kidney disease. Recently, sec63 has been found correlated with renal interstitial inflammation [13]. sec is the symbol of hsa_circ_0008925. However, it is still uncertain if urinary exosomal circRNAs, especially has_circ_0008925, have the potential to act as renal fibrosis biomarkers. In this study, we aimed to identify the potential candidate biomarkers of RF through the detection of urinary exosomes and further explore the relationship of urinary exosomal has_circ_0008925 with histological changes in glomerular disease patients with RF.

## 2. Methods

### 2.1. Cell Culture and Treatment

To determine if TGF-*β*1-treated HK-2 cell could secrete exosomal has_circ_0008925, HK-2 cells were cultured with Dulbecco's modified Eagle medium (DMEM)/F12 medium (Gibco) supplemented with 10% fetal bovine serum (FBS, Gibco, USA), 100 U/mL penicillin (Gibco), and 100 *μ*g/mL streptomycin (Gibco, USA) in a humidified incubator with 5% CO_2_ at 37°C. HK-2 cells were purchased from the National Collection of Authenticated Cell Cultures (Shanghai). The 1 × 10^7^ HK-2 cells were exposed to 100 ng/mL recombinant TGF-*β*1 protein (Sino Biological, China) for 24 and 48 hours. The cell supernatants were collected. After centrifugation in 300 g at 4°C for 10 min, the supernatants were collected and further centrifuged in 2000 g at 4°C for 20 min. Then, the supernatants were collected and centrifuged in 100000 g at 4°C for 70 min. The precipitates were collected as exosomes.

### 2.2. Exosomes from Cell Supernatants Identification

The exosomes from cell supernatants were identified by transmission electron microscope. In brief, a 10 *μ*L exosome sample was dropped on the copper net for precipitation for 1 min, and the filter paper absorbs the floating liquid. Then uranyl acetate 10 *μ*L is added to the copper net for precipitation for 1 min, and the floating liquid is absorbed by the filter paper and dried at room temperature for several minutes. The exosomes were imaged by transmission electron microscope (TEM, Hitachi, HT-7700) at 100 kV. Nanoparticle tracking analysis (NTA) (Particle Metrix, ZetaView) was performed to confirm the particle size. Isolated exosome samples were appropriately diluted using PBS to measure the particle size and concentration. NTA measurement was recorded and analyzed at 11 positions. The ZetaView system was calibrated using 110 nm polystyrene particles. Temperature was maintained around 23°C and 30°C. Western blot was performed to confirm the expression of exosomes specific marker CD9 and TSG101 using rabbit CD9 antibody, rabbit TSG101 antibody (ProteinTech, USA) and HRP-labeled goat anti-rabbit IgG (H+L) (Beyotime, China). *β*-Actin was used as a loading control (Biosharp, China).

### 2.3. Detection of hsa_circ_0008925 in Exosomes from Cell Supernatants

The whole RNA in exosomes was isolated using TRIzol-LS (Invitrogen, USA) according to the manufacturer's protocol. The concentration and purity of RNA were assessed using the relative absorbance ratio at 260/280 in a NanoDrop 2000 (Thermo, USA). The reverse transcription was performed using THE PrimeScript™ RT Reagent Kit (TAKARA, Japan) according to the manufacturer's protocol. RT-PCR was performed using the TB Green Premix Ex Taq Kit (TAKARA, Japan). The primers was as follows: hsa_circ_0008925 primers (sense: 5′-TTATGGCTGTCCTTGGGAGTTT-3′; antisense: 5′-GGTATTCTCGGTCTGTTTTGGA-3′) and U6 primers (sense: 5′-GCTTCGGCAGCACATATACTAAAAT-3′; antisense: 5′-CGCTTCACGAATTTGCGTGTCAT-3′). The hsa_circ_0008925 expression was normalized to U6 and calculated as 2^−*ΔΔ*Ct^.

### 2.4. Study Population

To determine if urinary exosomes displayed a high-level expression of hsa_circ_0008925 in glomerular disease patients, a total of 95 biopsy-proven glomerular disease patients were selected from the Department of Nephrology, Yijishan Hospital, Wannan Medical College. The pathological types of 95 glomerular disease patients concluded IgA nephropathy (*n* = 34), membranous nephropathy (*n* = 27), minimal-change glomerulonephritis (*n* = 16), focal segmental glomerulosclerosis (*n* = 2), diabetic nephropathy (*n* = 3), hypertensive nephropathy (*n* = 4), intracapillary proliferative glomerulonephritis (*n* = 1), mesangial proliferative glomerulonephritis (*n* = 3), membranoproliferative glomerulonephritis (*n* = 1), crescentic glomerulonephritis (*n* = 2), and lupus nephritis (*n* = 2). The exclusion criteria were as follows: patients younger than 18 years old or older than 80 years old; patients with chronic liver disease, urinary tract infection, cancer, or organ transplantation; glomerular disease patients with severe complications: cardiovascular disorder; or the use of steroids or immunosuppressive medications. The results of the laboratory examination were collected. Age- and gender-matched healthy volunteers (*n* = 54) also enrolled who were defined with the absence of abnormalities on a routine urinalysis and normal renal function (estimated glomerular filtration rate (eGFR) > 90 mL min/1.73 m^2^).

### 2.5. Collection of Urine Samples and Exosomes

The whole-stream early-morning urine specimens were collected after hospitalization. 100 mL urine sample was centrifuged at 3000 g for 30 min at 4°C. Then, the supernatants were centrifuged at 13500 g for 30 min at 4°C. The sediments were discarded, and the supernatants were centrifuged at 100000 g for 70 min at 4°C. The sediments were suspended in 100 *μ*L phosphate buffer saline (PBS) as urinary exosomes. Exosomes were identified as described above.

### 2.6. Detection of hsa_circ_0008925 in Urinary Exosomes

The RNA from exosomes was isolated as described above. The reverse transcription and RT-PCR were performed as described above.

### 2.7. Assessment of Renal Fibrosis

Analysis of renal fibrosis was performed on paraffin-embedded sections stained with periodic acid-Schiff and Masson trichrome. Serial 3 *μ*m sections were acquired from each paraffin block. Two experienced pathologists who were blinded to the results of molecular studies subjectively scored the severity of renal fibrosis. The degree of renal fibrosis for glomerular disease patients was evaluated, and the score of TIF and glomerular sclerosis performed on Masson-stained sections was calculated according to the previous study [3]. No fibrosis was considered 0 of the renal interstitium fibrosis. Mild-moderate fibrosis was considered ≤50%. Severe was considered >50%.

### 2.8. Western Blot

The protein in exosomes from cell supernatants and urine were collected using RIPA lysis buffer (Beyotime, China). Protein concentration was determined using a BCA kit (Beyotime, China). The CD9 and TSG101 protein expressions were determined by western blot. Rabbit anti-human CD9 antibody (ProteinTech, USA) and rabbit anti-human TSG101 antibody (ProteinTech, USA) were used. HRP-labeled goat anti-rabbit IgG (Beyotime, China) was used as the secondary antibody. *β*-Actin was used as a loading control (Biosharp, China).

### 2.9. Statistical Analysis

SPSS 17.0 was used for data analysis. The method of calculating the relative expression of circRNA was described in the previous study. Statistical comparison of different types of data, the correlation coefficient, logistic regression, and receiver operating characteristic (ROC) curves were calculated according to a previous study described [3]. Relative changes in gene expression were calculated using the *ΔΔ*Ct (threshold cycle) method: ΔCt = Ct gene of interest − Ct internal control, while ΔΔCt = Ct gene of interest − Ct internal control sample − Ct gene of interest − Ct internal control. Fold change = target gene expression level of sample/target gene expression level of control = 2^−ΔΔCt^. Normal distribution and equal variance data were expressed as mean ± standard deviation and compared using Student's *t*-test. Variance inequality or nonnormal distribution data were expressed as median (min, max). A Mann–Whitney test was used for variance inequality or nonnormal distribution data. Spearman's rank-order correlation coefficient was used to assess associations between gene expression levels and clinical parameters. Stepwise multivariate logistic regression analysis was used to assess the predictors for renal fibrosis. The diagnostic performance of biomarkers was evaluated using receiver operating characteristic (ROC) curves. The diagnostic threshold for maximum sensitivity and specificity was calculated. All *P* values were two-tailed, and *P* < 0.05 was considered statistically significant.

## 3. Results

### 3.1. Elevated Expression of hsa_circ_0008925 in Cell Supernatant-Derived Exosomes

**Figure 1:**
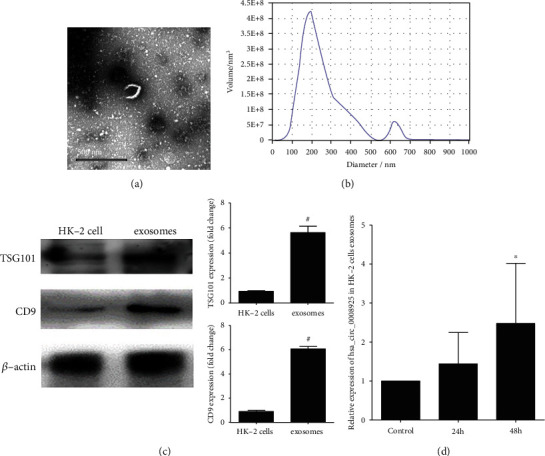
HK-2 cell-derived exosomes and hsa_circ_0008925 expression. (a) TEM image of exosomes. (b) NTA analysis of exosomes. (c) Western blot showed that exosomes from HK-2 cells expressed CD9 and TSG101. Histogram showed that the fold change of CD9 and TSG101 protein expression was normalized to *β*-actin (^#^*P* < 0.05 vs. HK-2 cells). (d) RT-PCR showed that coculture with 100 ng/mL for 48 h significantly increased the expression of hsa_circ_0008925 in HK-2 cell-derived exosomes (^∗^*P* < 0.001 vs. 24 h).

To identify the change of exosome release in TGF-*β*1-treated HK-2 cell, we isolated, characterized, and quantified exosomes from cell supernatants using transmission electron microscopy (TEM), western blotting (using CD9 and TSG101 as exosome markers), and ZetaView nanoparticle tracking analysis (NTA).  Figure 1(a) showed the TEM image of exosomes. The NTA is shown in Figure 1(b). Exosomes purified from cell culture supernatants showed the typical size and shape. The western blot exhibited that exosomes expressed specific protein markers CD9 and TSG101 compared to HK-2 cells (Figure 1(c)). RT-PCR showed that after coculture with 100 ng/mL TGF-*β*1 for 24 h and 48 h, the hsa_circ_0008925 expression in cell-derived exosomes was significantly increased compared to normal cultured (Figure 1(d)).

### 3.2. Identification of Urinary Exosomes from Healthy and Glomerular Disease Patients

**Figure 2:**
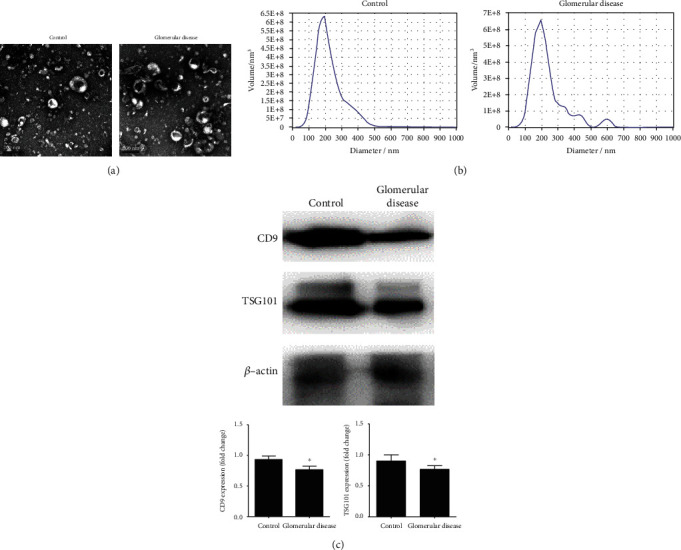
Urine-derived exosomes from healthy control and patients' identification. (a) TEM image of exosomes. (b) NTA analysis of exosomes. (c) Western blot showed that urinary exosomes expressed CD9 and TSG101. Histogram showed that the fold change of CD9 and TSG101 protein expression was normalized to *β*-actin (^∗^*P* > 0.05 vs. control).

The TEM images and ZetaView nanoparticle tracking analysis of urinary exosomes are shown in Figure 2. Exosomes purified from urine exhibited the typical size and shape. The western blot of CD9 and TSG101 (Figure 2(c)) identified that exosomes expressed specific protein markers CD9 and TSG101.

### 3.3. Baseline Characters of Patients

**Figure 3:**
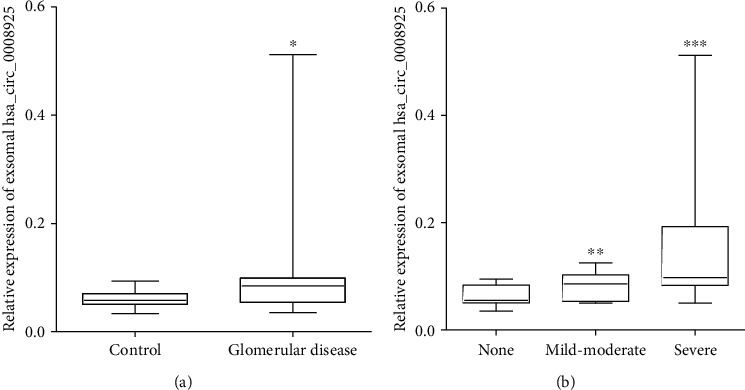
hsa_circ_0008925 expression in urinary exosomes from glomerular disease patients and healthy control. (a) Expression of hsa_circ_0008925 was significantly increased in glomerular disease patients compared to healthy controls. (b) In glomerular disease patients, urinary exosome hsa_circ_0008925 expression was significantly increased in mild-moderate and severe renal fibrosis patients compared to no fibrosis (^∗^*P* < 0.001 vs. control; ^∗∗^*P* = 0.002 vs. no fibrosis; ^∗∗∗^*P* = 0.0038 vs. mild-moderate).

The clinical characters of the involved subjects are shown in Table 1. There were no significant differences in age and gender between glomerular disease patients and controls. The glomerular disease patients showed high levels of Scr, BUN, and cystatin C and low estimated glomerular filtration rate (eGFR) compared with controls. eGFR was calculated by modified MDRD equations [14]. The relative expression level of hsa_circ_0008925 was significantly increased in the glomerular disease patients (median expression 0.085 in glomerular disease vs. 0.058 in healthy controls, *P* < 0.001 vs. control, Figure 3(a)).

The 95 glomerular disease patients included 28 patients without renal fibrosis, 39 patients with mild-moderate renal fibrosis, and 28 patients with severe renal fibrosis. As shown in Table 2, there were no significant differences in age, gender, and 24 h proteinuria among these 3 groups. The relative expression level of urinary exosomal hsa_circ_0008925 was significantly higher in the mild-moderate group and severe group compared to the no fibrosis group (Median expression 0.086 in mild-moderate vs. 0.055 in the no fibrosis group, *P* = 0.002; mild-moderate vs. 0.098 in severe, *P* = 0.038; Figure 3(b)).

### 3.4. Correlation between Urinary Exosomal hsa_circ_0008925 and Clinical Parameters as well as Pathological Parameters

**Figure 4:**
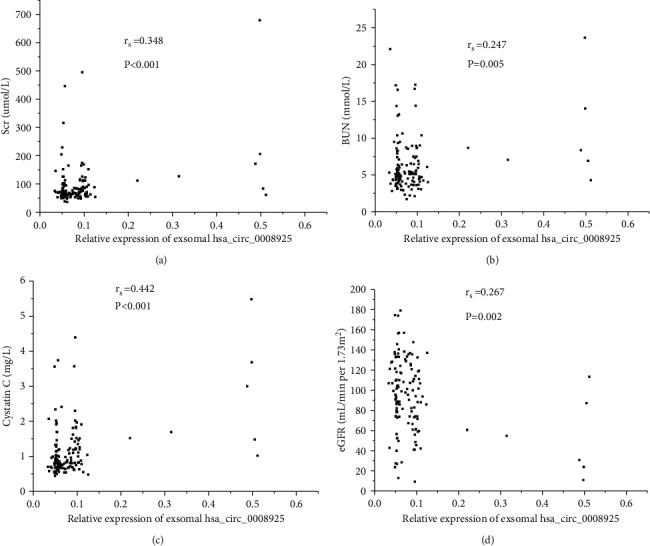
Correlation between urinary exosomal hsa_circ_0008925 expression and kidney function. (a) Spearman's correlation between hsa_circ_0008925 and Scr (*r*_s_ = 0.348, *P* < 0.001). (b) Spearman's correlation between hsa_circ_0008925 and BUN (*r*_s_ = 0.247, *P* = 0.005). (c) Spearman's correlation between hsa_circ_0008925 and cystatin C (*r*_s_ = 0.442, *P* < 0.001). (d) Spearman's correlation between hsa_circ_0008925 and eGFR (*r*_s_ = −0.267, *P* = 0.002).

**Figure 5:**
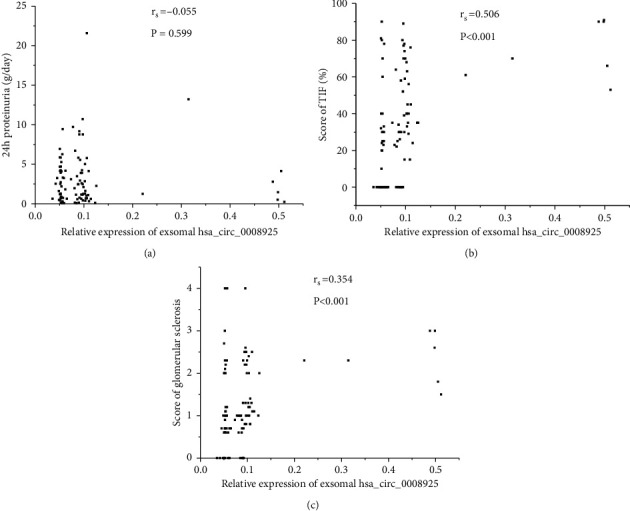
Correlation between urinary exosomal hsa_circ_0036649 expression and 24 h proteinuria, renal fibrosis pathological score. (a) Spearman's correlation between hsa_circ_0008925 and 24 h proteinuria (*r*_s_ = −0.055, *P* = 0.599). (b) Spearman's correlation between hsa_circ_0008925 and the score of TIF (*r*_s_ = 0.506, *P* < 0.001). (c) Spearman's correlation between hsa_circ_0008925 and the score of glomerular sclerosis (*r*_s_ = 0.354, *P* < 0.001).

Urinary exosomal hsa_circ_0008925 correlated with Scr (*r*_s_ = 0.348, *P* < 0.001), BUN (*r*_s_ = −0.215, *P* = 0.005), cystatin C (*r*_s_ = 0.442, *P* < 0.001), and eGFR (*r*_s_ = −0.267, *P* = 0.002; Figure 4). In glomerular disease patients, urinary exosomal hsa_circ_0008925 correlated with the score of TIF (*r*_s_ = 0.506, *P* < 0.001) and the score of glomerular sclerosis (*r*_s_ = 0.354, *P* < 0.001; Figure 5). However, urinary exosomal hsa_circ_0008925 did not correlate with 24 h proteinuria (*r*_s_ = −0.055, *P* = 0.599).

The stepwise multivariate logistic regression analysis further showed that the relative expression of urinary exosomal hsa_circ_0008925 significantly correlated with renal fibrosis (Table 3, OR: 1.517; 95% CI: 1.178-1.954; *P* = 0.001). In addition, Scr, BUN, cystatin C, eGFR, and 24 h proteinuria had no statistical significance in the correlation with renal fibrosis by the stepwise multivariate logistic regression analysis (Table 3).

### 3.5. Diagnostic Value of Urinary Exosomal hsa_circ_0008925 for Renal Fibrosis

**Figure 6:**
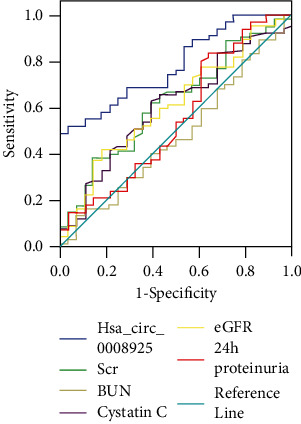
The receiver operating characteristic (ROC) curve showed the diagnostic value of the urinary exosomal hsa_circ_0008925 for renal fibrosis. ROC curve showed the urine exosomes hsa_circ_0008925-distinguished renal fibrosis (AUC of 0.782, 95% CI: 0.690-0.874; *P* < 0.001).

The results showed that urinary exosomal hsa_circ_0008925 could effectively distinguish renal fibrosis from no fibrosis, with the largest AUC of 0.782 (95% CI: 0.690-0.874; *P* < 0.001) higher than that of Scr (AUC of 0.625; 95% CI: 0.504-0.760; *P* = 0.055), BUN (AUC of 0.479; 95% CI: 0.351-0.606; *P* = 0.744), cystatin C (AUC of 0.606; 95% CI: 0.484-0.729; *P* = 0.103), eGFR (AUC of 0.612; 95% CI: 0.489-0.734; *P* = 0.087), and 24 h proteinuria (AUC of 0.555; 95% CI: 0.421-0.688; *P* = 0.403). Urinary exosomal hsa_circ_0008925 displayed a sensitivity of 52.2% and specificity of 96.4% at the optimal cut-off value of 0.093 (Figure 6).

## 4. Discussion

In this study, we found that tubular epithelial cell-derived exosomes contained a high level of hsa_circ_0008925 in the renal fibrosis model in vitro. In addition, we found that the urinary exosomal hsa_circ_0008925 expression level was increased in glomerular disease patients. Furthermore, within glomerular disease patients, patients with renal fibrosis showed a higher urinary exosomal hsa_circ_0008925 expression level than patients with no fibrosis, which suggested the potential of urinary exosomal hsa_circ_0008925 to become a noninvasive biomarker for renal fibrosis.

Noninvasive and repeatable detection of renal fibrosis is the key for early monitoring and prevention of glomerular disease progression. Additionally, biomarkers often play an important role in the occurrence and development of diseases and are major molecules to reveal the pathogenesis of diseases. However, renal biopsy is incapable of the advantages of being noninvasive and repeatable. Noninvasive markers and detection methods of renal fibrosis are necessary in clinical practice. In 2001, Li et al. first established a noninvasive approach to diagnose acute renal rejection of allografts by isolating and quantifying RNA of specific genes in urine sediments [5]. Subsequently, many studies found that the RNA contained in urinary sediment cells is related to a variety of kidney diseases and has the application value of biomarkers [3, 14–17]. Urinary sediment contains different types of cells, including renal intrinsic cells. The difference in mRNA expression in urinary sediment cannot accurately reflect the state of renal disease.

Pisitkun et al. first found exosomes in urine [10]. Urine exosomes are produced by the fusion of MVB with renal epithelial cells which can reflect the pathological changes of the kidney [18]. Previous studies have found that protein, mRNA, and miRNA in urinary exosomes can be used as biomarkers for the diagnosis of CKD [4, 19, 20]. Exosomes contained abundant circRNAs [21]. Ma et al. reported that 89 circRNAs were significantly differentially expressed in idiopathic membranous nephropathy patients' urine exosomes and MUC3A could be considered a potential diagnostic biomarker of idiopathic membranous nephropathy [9]. In our study, we found that HK-2 cell-derived exosomes contained a high level of hsa_circ_0008925 in the fibrotic microenvironment in vitro. That strongly indicated that in renal fibrosis, renal tubular epithelial cells secrete a large number of circRNAs carried by exosomes. Urinary exosomal hsa_circ_0008925 expression level correlated with renal function and pathological changes, which may show the potential of the ability to diagnose renal fibrosis.

Tubular epithelial cells play an important role in renal fibrosis. A previous study showed that the metabolic state of tubular epithelial cells and excessive accumulation of the extracellular matrix are important factors which may influence renal fibrosis [22, 23]. There are a few studies on whether circular RNA in tubular epithelial cells is involved in renal fibrosis. Peng et al. found that the circRNA_010383 expression level was markedly downregulated in tubular epithelial cells cultured in high-glucose conditions. Loss of circRNA_010383 promoted proteinuria and renal fibrosis in DN by acting as a sponge for miRNA-135a [24]. Wen et al. reported that circACTR2 regulated high glucose-induced pyroptosis, inflammation, and fibrosis in proximal tubular cells. circACTR2 was defined as a novel circular RNA-regulated high glucose-induced fibrosis [25]. Cui et al. revealed that circZNF609 is involved in the pathogenesis of focal segmental glomerulosclerosis [26]. Xu et al. further found that circEIF4G2 aggravates renal fibrosis in mouse models and rat cell lines [27]. These researches indicated that in CKD that progressed to renal fibrosis, circRNAs played a key role in the progression of the disease. In our research, we first suggested that the expression level of hsa_circ_0008925 increased in TGF-*β*1-cultured HK-2 cell-derived exosomes. The elevated level of hsa_circ_0008925 in urinary exosomes was probably from tubular epithelial cells. So far, there was no relevant research that revealed the biological function of hsa_circ_0008925. hsa_circ_0008925 originated from chr6:108222573-108246136, and the gene symbol was sec63. Ishikawa et al. found that the loss of sec63 promoted spliced XBP1 which alleviated renal interstitial inflammation [13]. In this study, our data strongly suggested that hsa_circ_0008925 participated in the progression of renal fibrosis and indicated that hsa_circ_0008925 might be used as a noninvasive biomarker of renal fibrosis.

Our study also has some limitations. First, this study did not focus on the specific pathological types of glomerular disease. Whether the urinary exosomal hsa_circ_0008925 can be used as renal fibrosis biomarkers in different pathological types still needs to be further determined by large sample size research. Second, subsequent cell and animal studies are needed in the future research to determine the mechanism of hsa_circ_0008925 in renal fibrosis. Additionally, whether hsa_circ_0008925 will affect the prognosis of patients with renal fibrosis is uncertain and still needs to be further studied.

## 5. Conclusion

Patients with renal fibrosis showed a higher urinary exosomal hsa_circ_0008925 expression level than patients with no fibrosis, which suggested the potential of urinary exosomal hsa_circ_0008925 to become a noninvasive biomarker for renal fibrosis.

## Figures and Tables

**Table 1:** Clinical characteristics of glomerular disease patients and healthy controls.

	Glomerular disease (*n* = 95)	Control (*n* = 34)	*P* value
Age (years)	44.5 ± 14.6	43.6 ± 15.5	0.756
Gender (male/female)	51/44	25/9	0.067
24 h proteinuria (g/day)	2.190 (0.080-21.580)	/	/
Scr (mmol/L)	107.9 ± 92.0	62.3 ± 7.0	0.005
BUN (mmol/L)	7.1 ± 4.1	4.4 ± 1.1	<0.001
Cystatin C (mg/L)	1.39 ± 0.86	0.67 ± 0.10	<0.001
eGFR (mL/min per 1.73 m^2^)	84.1 ± 36.0	121.0 ± 22.8	<0.001
Score of TIF	30 (0-91)	/	/

Score of glomerular sclerosis	1.0 (0-4.0)	/	/

Exosomal hsa_circ_0008925	0.085 (0.035-0.511)	0.058 (0.033-0.093)	/ 0.001

Abbreviations: Scr: serum creatinine; eGFR: estimated glomerular filtration rate; BUN: blood urea nitrogen.

**Table 2:** Clinical and pathological characteristics of glomerular disease patients.

	None (*n* = 28)	Mild-moderate (*n* = 39)	Severe (*n* = 28)	*P* value
Age (y)	42.9 ± 18.2	41.7 ± 12.2	49.9 ± 12.6	0.061
Sex (M/F)	11/17	21/18	19/9	0.100
Scr (*μ*mol/L)	83.5 ± 37.1	88.6 ± 73.0	159.2 ± 129.4	0.002
BUN (mmol/L)	7.1 ± 4.2	5.6 ± 3.0	9.1 ± 4.7	0.002
Cystatin C (mg/L)	1.19 ± 0.63	1.06 ± 0.54	2.05 ± 1.07	<0.001
eGFR (mL/min per 1.73 m^2^)	94.2 ± 35.8	98.6 ± 31.1	54.0 ± 22.8	<0.001
24 h proteinuria (g/day)	2.130 (0.080-9.160)	1.290 (0.100-21.580)	2.810 (0.220-13.210)	0.227
hsa_circ_0008925	0.055 (0.035-0.095)	0.086 (0.051-0.125)	0.098 (0.050-0.512)	<0.001
Score of TIF (%)	0	30 (10-45)	70 (52-91)	<0.001
Score of glomerular sclerosis	0.6 (0-1.0)	1.0 (0-2.4)	2.3 (1.0-4.0)	<0.001

Abbreviations: Scr: serum creatinine; eGFR: estimated glomerular filtration rate; BUN: blood urea nitrogen.

**Table 3:** Multivariate logistic regression analysis of selected variables for renal fibrosis.

	OR	95% CI	*P* value
hsa_circ_0008925	1.517	1.178-1.954	0.001
Scr	1.027	0.993-1.063	0.116
BUN	0.800	0.611-1.046	0.103
Cystatin C	0.660	0.104-4.189	0.660
eGFR	0.996	0.969-1.024	0.784
24 h proteinuria	1.093	0.889-1.343	0.400

Abbreviations: Scr: serum creatinine; eGFR: estimated glomerular filtration rate; BUN: blood urea nitrogen; OR: odds ratio; CI: confidence interval.

## Data Availability

The datasets used and/or analyzed in the current study are available from the corresponding author on reasonable request.
